# Goodpasture syndrome in pregnancy without renal involvement: A case report

**DOI:** 10.22088/cjim.13.2.442

**Published:** 2022

**Authors:** Taghi Riahi, Laily Najafi, Omolbanin Asadi Ghadikolaei, Mohammad Hadi Tajik Jalayeri

**Affiliations:** 1 Hazrate Rasool Hospital, Iran University of Medical Sciences (IUMS), Tehran, Iran; 2 Endocrine Research Center, Institute of Endocrinology and Metabolism, Iran University of Medical Sciences (IUMS), Tehran, Iran; 3 Masih Daneshvari hospital, Shahid Beheshti University of Medical Sciences, Tehran, Iran

**Keywords:** Goodpasture syndrome, Pregnancy, Respiratory failure, renal involvement, Case report

## Abstract

**Background:**

Goodpasture syndrome (GPS) rarely affects parturients which may quickly result in severe pulmonary and renal damage with significant fetomaternal morbidity.

**Case Presentation:**

A 35-year-old white multiparous lady, presented with acute progressive respiratory failure at 32^th^ gestational age. She had fever, cough, severe dyspnea and lately hemoptysis and severe hypoxia with bilateral alveolar opacity in chest imaging, with no response to broad spectrum antibiotic. GPS diagnosis was confirmed by high anti- glomerular basement membrane (anti GBM) titer, without the similar history in the past parities. High dose intravenous methylprednisolone ended to dramatic clinical response. She was maintained on glucocorticoids for five weeks before the successful delivery of a live healthy fetus at 39 Weeks.

**Conclusion:**

This study demonstrated a successful pregnancy outcome which was achieved in the present GPS parturient with a careful antepartum care involving maternal-fetal status by serial pulmonary, renal monitoring and special treatment of disease.

Goodpasture's syndrome (GPS) is a rare but serious autoimmune disease in which antibodies attack the basement membrane in lungs and kidneys, leading to pulmonary hemorrhage and acute glomerulonephritis (1). The incidence of GPS is <1case/1000000, and the precise cause is unknown (2), but it is believed in addition to genetic predisposition (3), the exposure to organic solvents or hydrocarbons, tobacco smoke, metallic dust, high oxygen environments or viral and bacterial infections, sepsis and cocaine abuse may trigger the disease. The symptoms of this condition (myalgia, arthralgia, fever and etc.) usually occur gradually over several months or years (3). In certain cases, however, the signs and symptoms develop quickly over just a few days. Early diagnosis of the disorder is very essential for a good prognosis. Therapeutic modalities remain controversial and varied among clinical experts. 

Five-year survival is more than 80% in and fewer than 30% of affected individuals require long-term dialysis (4). The occurrence of GPS during prenatal period is very rare. This uncommon pregnancy complication is associated with significant feto-maternal mortality and morbidity. The management of GPS during prenatal period requires intensive care and multidisciplinary cooperation (7). The mentioned syndrome mostly occurs in pregnancy with late diagnosis that may produce complications for both mother and fetus (8). The current management modalities constitute difficult antepartum problem and regarding aforementioned treatments, the parturients have poor prognosis. We describe a case of anti-GBM antibody alveolar hemorrhage in the last trimester of pregnancy with rapid onset respiratory complaint.

## Case Presentation

A 35 years-old multiparous woman at 32 weeks of gestation (WOG) was presented with acute progressive respiratory complaints for five days prior to admission. She had fever, cough, dyspnea and transient hemoptysis that had been admitted to 5^th^ Azar hospital (Gorgan) as pneumonia, but her progressive respiratory distress caused her to transfer to intensive care unit. No significant past medical history was mentioned. Merely she mentioned mild transient arthralgia from time to time in the past few weeks without arthritis that improved spontaneously. She did not report any exposure to chemical materials, alcohol or smoke. On physical examination, she was alert, febrile, tachypneic and tachycardic. Her blood pressure and rest of system examinations were within normal limits. At auscultation, there was a bilateral crackle. Laboratory study revealed mild hypochromic microcytic anemia and bandemia. ESR, liver function tests, and urine analysis were in normal range. Sputum culture and smear were negative for bacteriologic study, only hemosiderin-laden macrophages were reported in smear analysis, so the alveolar hemorrhage is demonstrated for the present case. Obstetric examination and sonography were normal. Primary laboratory findings on the first hospital admission are demonstrated in table 1.

**Table 1:** Laboratory findings on the first hospital admission

**Laboratory findings**	**Values**	**Normal range**
**Blood** Hemoglobin (Hb) Total white blood cell count Neutrophils Eosinophil Lymphocytes Band cell Erythrocyte sedimentation rate (ESR) Platelet count High sensitivity C-reactive protein (Hs-CRP) Blood Urea Nitrogen (BUN) Serum Creatinine Serum Sodium Serum Potassium Blood culture	10.7g/dl 10600 /mm^3^ 84% 2% 10% 4% 12 mm/hr 204000 /mm^3^ 0.8 mg/l 9 mg/dl 0.7 mg/dl 142mEq/l 3.8mEq/l Neg^a^	11.5-16.5 4000-11000 40-60% 1-3% 20-40% 0-5% 1-25 150000-400000 - 7-20 0.7-1.4 136-145 3.5-5.1
**Urine** Albumin Red blood cells (RBCs) White blood cells (WBCs) Blood Cast	Trace 0-1 0-1 Neg^a^ Neg^a^	

^a^ *Neg: negative*

**Figure 1(A, B):**
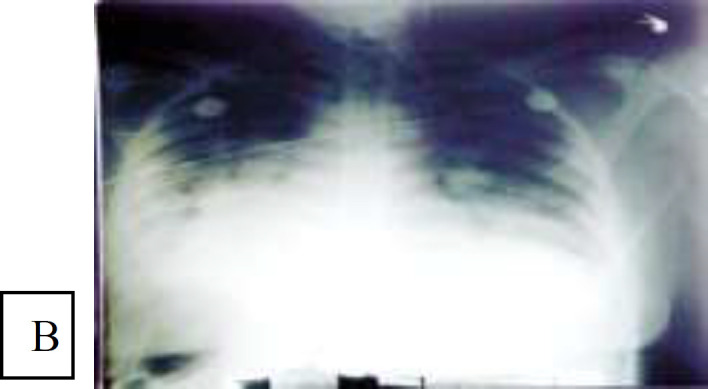
Chest x-rays showing abnormal white patches associated with lung hemorrhage by 3 days interval. The second one (B) showed significant progression of past lesions which dictated bad prognosis

First chest x-ray (CXR) was performed with protective shield, which is shown bilateral, symmetrical, perihilar and basilar patchy consolidation (figure 1A). Transthoracic echocardiogram was done for the patient and the results were normal. She was treated for severe diffuse pneumonia with meropenem, vancomycin, azithromycin and oseltamivir. After 72 hours, despite mentioned management in addition to oxygen and fluid therapy, no significant improvement was observed in patient's condition. Although fever stopped but respiratory distress continued and the patient still suffered from productive coughs and also hemoptysis was added to patient's symptoms that mandated supplementary studies. The second CXR was repeated in figure 1B. 

**Figure 2:**
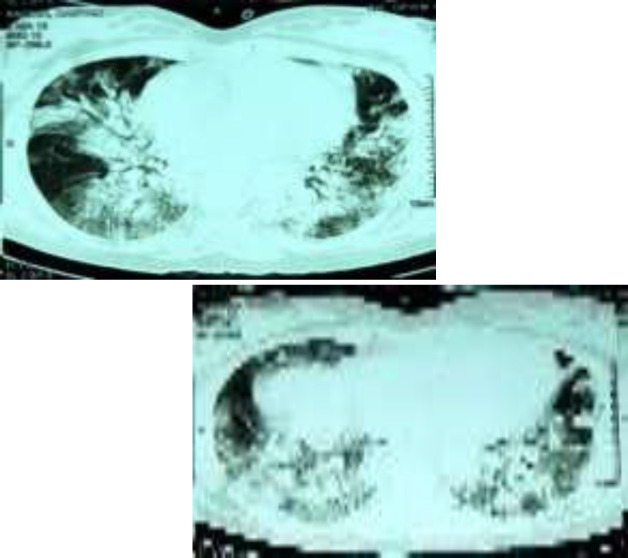
CT scan showing disseminated heterogenic alveolar consolidation

The physicians decided to perform lung computerized tomography (CT) scan with lead cover in addition to complementary lab data. The CT scan is showed in figure 2. In lung CT scan, disseminated heterogenic alveolar consolidation mostly in middle and lower parts was evident. 

Regarding to symptoms, disease trend and paraclinical evaluations; to discriminate between possible differential diagnosis (5), the complementary laboratory findings are performed in table 2.

**Table 2:** Complementary laboratory findings

**Laboratory variable**	**Patient's value**	**Normal value**
P-ANCA^ a^	4.9	Neg†<20 Pos‡>20
C-ANCA^b^	5.4	Neg<20 Pos>20
ANA^c^	0.8	Neg<10 Pos>10
Anti CCP^d^	2	Neg<6.25 Pos>6.25
Anti-GBM^e^	90.2	Neg<8 Borderline: 8-12 Pos>12

^a^ **P-ANCA: Perinuclear Anti-Neutrophil Cytoplasmic Antibodies,** ^b^ **C-ANCA: Cytoplasmic antineutrophil cytoplasmic antibodies ,** ^c^ **ANA: Antinuclear antibodies ,** ^d^ **Anti CCP :Anti–cyclic citrullinated peptide ,** ^e^ **anti-GBM: Anti-glomerular basement membrane**

**†Neg: Negative, ‡Pos: Positive**

The complementary laboratory findings were as follows: serum complement levels (C3 and C4), P-ANCA, C-ANCA, ANA and Anti CCP were normal, the H1N1 PCR was negative. The serum anti-GBM antibody was double checked by ELIZA, and it got laboratory-confirmed positive high-titer results. Due to all clinical and paraclinical evaluations (CT scan pattern and high anti-GBM titer (the most sensitive and specific test for GPS) (3, 6)) GPS was labeled. There was no confirmed family history of GPS. No renal involvement was detected for her, and she was managed by intravenous methylprednisolone pulse therapy (1 g/daily) for 3 days. The patient responded quickly and improved dramatically after the first dosage of steroid and all signs and symptoms improved. After a week she was discharged with oral prednisolone maintenance. No plasmapheresis was needed. She visited weekly for respiratory complaint and fetal health for the rest of pregnancy. The CXR and CT scan was not performed at this point due to the patient’s worries regarding fetus safety.

She delivered a normal birth weight (BW) neonate at 39 WOG by Cesarean section (CS). The neonatal apgar score of 9 at 5 minutes was documented. The neonate seemed well and healthy, requiring only routine supportive care. Maternal anti-GBM antibody titer became negative after delivery and fortunately her response was so quick. In two years regular follow-up; she was well without any respiratory complications. 

## Discussion

GPS syndrome rarely presents in pregnancy with limited data for its incidence and prevalence, and may result in significant adverse feto-maternal outcomes (7, 8). It has been shown that the autoantibodies are capable of binding to tissues other than the kidney’s tissue, such as the placenta, which may have complication for both mother and baby (7). This antibody is found in more than 90% of GPS patients. 60-80% of the patients’ lungs and kidneys are both involved. While 5-10% of the patients only one lung is involved and in the rest, the kidney is involved solely (2, 9). Lack of renal involvement in our patient can either be due to early diagnosis, or to partnership in the group of 5-10% of solitary lung involvement. 

It is more common in males with peak age ranges of 20-30 and 60-70 years and it affects both sexes (3). In the review of the previous pregnant cases since 1986 till the present time; the mean age of the patients was 27.6 (3, 9) and 29.3±2.5 years old (1). Presentation of this syndrome in the second trimester was more common (12.5±5.9 weeks) (1, 8, 10, 11); except the cases of Adnan and Deubner (5, 12), Wells and Friend (2, 7), Yankowitz (6) and Hatfield (3) which presented at 1^st^ trimester, 3^rd^ trimester, 3 months and 13 years prior to pregnancy, respectively. Most of the parturients were primiparous (75%) and 25% of them were multiparous. Past medical history was positive in two cases which were presented by IgA nephropathy (10) and hypertension(3, 9). Our multiparous case was near to the common age range (1), and presented at 3^rd^ trimester. 

The disease trend is more progressive in younger groups and presents as cough, hemoptysis, hemoglobin decrement, dyspnea, fever and hematuria (2, 8-10). Hypertension and proteinuria are the most common pathological findings in parturients (1-3, 8, 9). Clinical condition of patients with pulmonary hemorrhage is much better than elderly patients with chronic kidney involvement. Due to high mortality rate in GPS, early diagnosis and treatment modalities are of special importance and improve the prognosis. In Yankowitz's (6) and Hatfield's (3) studies, the diagnosis was established prior to conception with positive anti-GBM antibody and positive renal biopsy. The anti-GBM antibody titer tapered and became negative pre-pregnancy (3) and during pregnancy (6). The anti-GBM antibody was in borderline range during pregnancy (12) and negative (13) in these two cases but got positive postpartum (12). In the rest of the cases, we had detected high titers of anti-GBM antibody (8-10). Special antepartum diseases such as gestational diabetes mellitus(8, 9), preeclampsia (2, 7, 8, 12), HELLP syndrome (3) and pneumocystis carinii were presented (9)and observed.

The antepartum GPS management requires intensive care and multidisciplinary medical team (1). Treatment includes removal of anti-GBM from the circulation, regulation and inhibition of inflammatory response and prevention of antibody production. The major mainstay treatment for GPS is plasmapheresis (10) to the removal of anti-GBM. Another treatment that should be used for these patients were immunosuppressants, especially cyclophosphamide, rituximab, azathioprine and prednisone to prevent the formation of new anti-GBM antibodies to further damage the kidneys and lungs (10). These patients had taken necessary antepartum treatment (45%) (8, 9, 13), or after pregnancy (34%) (10, 12, 14) and in two cases pre-pregnancy (3, 6). Plasmapheresis, cyclophosphamide and methylprednisolone were prescribed in most of the cases (3, 8-10, 12-14), also aspirin and azathioprine were used for two cases (9). The present case was not treated with typical treatments such as plasmapheresis, immunosuppressant medications (cyclophosphamide, rituximab azathioprine) which distinguishes our case from the others. Third trimester presentation and multiparous may affect the solitary pulmonary involvement, which may guide to good prognosis. At postpartum, the anti-GBM antibody titer fell and became undetectable. She recovered completely after corticosteroid therapy which inhibits the formation of immune antibodies. 

The anti-GBM antibody became negative after treatment and hemodialysis in Nair’s study (10), although some cases remained dialysis-dependent postpartum (3, 9, 12-14) and three of them underwent successful renal transplantation (3, 12, 14). Some etiologies for rapid recovery of GPS were early pregnancy termination (5), higher gravidity (12), and the placental removal (11). The majority of terminations were done prior to term delivery to prevent worsening the adverse fetomaternal outcomes(4, 10). Vaginal delivery (3, 9, 10, 12, 14) was dominant (67%) compared by CS. The outcomes in two pregnancies were healthy and normal BW and size (6, 12), four cases delivered healthy and small for gestational age neonates(3, 9, 13, 15), with the mean of 34.2 WOG and two cases had intrauterine growth restriction (IUGR) neonates (8, 9). Three cases were aborted before 20^th^ WOG (5, 11), one delivered a stillbirth fetus at 28 WOG (14) and another was therapeutically terminated at 15 WOG (10). In Vasiliou's study, a low BW and IUGR neonate at 26 WOG was delivered but some complications developed in the mentioned case (massive intraventicular hemorrhage, neurodevelopmental delay and so on) (8). Anti-GBM antibodies were positive in two newborns without renal or pulmonary involvements (9). The present case delivered a full-term, normal BW neonate by CS. The neonatal anti-GBM level was negative and undetectable. 

According to lack of disease progression, a favorable outcome may be possible, due to sufficient time to complete the duration of pregnancy. The role of pregnancy in initiating or in the recovery of GPS is questionable and needs complementary studies. The present case was advised not to smoke and avoid being a passive smoker. In addition, appropriate contraceptive, exercise and stress reduction were mentioned. Thus, regular and long-term follow-up was recommended to her.
